# Self-Cleavage of Human Chloride Channel Accessory 2 Causes a Conformational Shift That Depends on Membrane Anchorage and Is Required for Its Regulation of Store-Operated Calcium Entry

**DOI:** 10.3390/biomedicines11112915

**Published:** 2023-10-28

**Authors:** Grace T. Ramena, Aarushi Sharma, Yan Chang, Zui Pan, Randolph C. Elble

**Affiliations:** 1 Department of Medical Microbiology, Immunology, and Cell Biology, Southern Illinois University School of Medicine, Springfield, IL 62702, USA; ramenag@uapb.edu; 2 Department of Pharmacology and Simmons Cancer Institute, Southern Illinois University School of Medicine, Springfield, IL 62702, USA; sharma.aarushi1821@gmail.com; 3 Department of Graduate Nursing, College of Nursing and Health Innovation, The University of Texas at Arlington, Arlington, TX 76010, USA; yan.chang@uta.edu (Y.C.); zui.pan@uta.edu (Z.P.); 4 Bone and Muscle Research Center, College of Nursing and Health Innovation, The University of Texas at Arlington, Arlington, TX 76010, USA; 5 Department of Kinesiology, College of Nursing and Health Innovation, The University of Texas at Arlington, Arlington, TX 76010, USA

**Keywords:** CLCA2, metalloprotease, self-cleavage, SOCE

## Abstract

Human CLCA2 regulates store-operated calcium entry (SOCE) by interacting with Orai1 and STIM1. It is expressed as a 943aa type I transmembrane protein that is cleaved at amino acid 708 to produce a diffusible 100 kDa product. The N-terminal ectodomain contains a hydrolase-like subdomain with a conserved HEXXH zinc-binding motif that is proposed to cleave the precursor autoproteolytically. Here, we tested this hypothesis and its link to SOCE. We first studied the conditions for autocleavage in isolated membranes and then in a purified protein system. Cleavage was zinc-dependent and abolished by mutation of the E in the HEXXH motif to Q, E165Q. Cleavage efficiency increased with CLCA2 concentration, implying that it occurs in trans. Accordingly, the E165Q mutant was cleaved by co-transfected wildtype CLCA2. Moreover, CLCA2 precursors with different epitope tags co-immunoprecipitated. In a membrane-free system utilizing immunopurified protease and target, no cleavage occurred unless the target was first denatured, implying that membranes provide essential structural or conformational cues. Unexpectedly, cleavage caused a conformational shift: an N-terminal antibody that immunoprecipitated the precursor failed to precipitate the N-terminal product unless the product was first denatured with an ionic detergent. The E165Q mutation abolished the stimulation of SOCE caused by wildtype CLCA2, establishing that the metalloprotease activity is required for this regulatory function.

## 1. Introduction

Human Chloride Channel Accessory 2, CLCA2, is a 943 amino acid type I transmembrane protein that plays important roles in the differentiation and stress response of mammary epithelial cells and epidermal keratinocytes [1,2,3,4]. CLCA2 is so named because it is a member of a protein family that regulates calcium-activated chloride channels [5,6]. Among its functional interactions, it forms a complex with the regulators of store-operated calcium entry, Orai-1 and STIM-1, to augment cytosolic Ca^2+^, which in turn activates the calcium-activated chloride channel TMEM16A [7]. 

**Figure 1:**
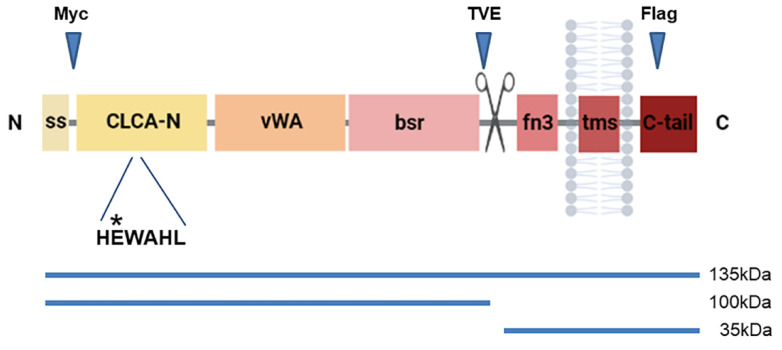
Schematic representation of CLCA2 protein structure. The locations of the TVE20, Myc tag, and Flag tag epitopes relative to the cleavage site (scissors) are indicated. ss, signal sequence; CLCA-N, metalloprotease domain; VWA, von Willebrand A domain; bsr, beta sheet domain; fn3, fibronectin-3-like domain; tms, transmembrane segment; C-tail, cytoplasmic tail. Relative location of the zinc-binding HEWAHL motif is indicated; asterisk, site of the E165Q substitution. Lower, apparent sizes of precursor and cleavage products.

The CLCA gene family arose in placozoans, perhaps the first multicellular organisms to develop epithelial tissues with organized cell–cell junctions [8]. In mammals, CLCAs comprise four subfamilies, three of which are conserved in humans: CLCA1, CLCA2, and CLCA4 [9]. This protein family is distinguished by the juxtaposition of a distinct metalloprotease domain termed CLCA-N; a VWA (von Willebrand Factor A) domain; a beta sheet domain, BSR; a fibronectin III domain; and the capacity to self-cleave near amino acid 700 [10] (Figure 1). The CLCA-N domain contains an HExxH metal-binding motif that is characteristic of metalloproteases. Mutation of the glutamate to glutamine abolished cleavage of the CLCA1 precursor [10]. This result was replicated by other groups with homologs from other species [11,12,13]. VWA domains are generally involved in protein–protein interactions and are conserved in all eukaryotes [14]. This domain is also coordinated by a metal-binding motif called the MIDAS box (metal ion-dependent adhesion site). VWA domains may undergo a conformational change upon ligand binding [15]. 

Of the human family members, CLCA1 is the best studied because of its well-established role in modulating mucus in airways and the gut in response to cytokines, in addition to its role in asthma [16,17,18]. Using isolated membranes from transfected cells, Bothe and co-workers established that CLCA1 cleavage was dependent on zinc and was intermolecular [11]. Later studies with affinity-purified protein confirmed these results and identified a consensus cleavage site [13]. The latter study also established that cleavage was functionally important, being essential for the regulation of a calcium-activated chloride channel [13]. 

Unlike CLCA1, CLCA2 has a C-terminal transmembrane segment that anchors it to the plasma membrane [1]. In a previous study, we established that a CLCA2 precursor is cleaved at the cell surface to produce a heavily glycosylated N-terminal ectodomain that remains associated with the membrane-anchored C-terminal product until released by a still unknown trigger [1]. The metalloprotease activity of CLCA2 has not been investigated, nor has the significance of cleavage to CLCA2 function.

In this study, we show that, like CLCA1, CLCA2 cleaves itself by intermolecular proteolysis that depends on zinc and a wildtype HExxH sequence. Unlike CLCA1, it also requires membrane association. Moreover, we show evidence that cleavage causes a conformational shift in the ectodomain. Mutation of the zinc-binding site not only prevents cleavage but abolishes the regulation of SOCE, showing a link between cleavage and function.

## 2. Materials and Methods

### 2.1. Cell Culture 

HEK293 and HEK293T cells were obtained from ATCC. HEK293 was used to establish stable cell lines, while HEK293T was used in experiments involving transient transfection. All cells were grown in DMEM (Corning, NY, USA) with 10% FBS (Gemini, CA, USA) [2]. 

### 2.2. Lentiviral Transduction 

HEK293 cells were transduced with pLex lentivirus (ThermoFisher, Waltham, MS, USA) expressing wildtype or mutant human CLCA2 labeled with a Flag epitope tag [19] from a CMV promoter and subjected to puromycin (Fisher, Waltham, MS, USA) selection, 1 microgram/mL. Viral packaging was performed as described [2]. 

### 2.3. Immunoblot and Antibodies 

Antibody TVE20 was raised against the human CLCA2 peptide TVEPETGDPVTLRLLDDGAG (aa643–663) in rabbits and affinity-purified over an antipeptide column (Proteintech Group, Rosemont, IL, USA). Anti-Flag mAb M2 was from Agilent (Santa Clara, CA, USA); anti-Myc mAb 9E10.3 was from Neomarkers; and anti-actin 125-ACT was from PhosphoSolutions (San Francisco, CA, USA). Secondary antibodies were tagged with IR680 or IR800 (Licor, Lincoln, NE, USA), and blots were scanned and quantified fluorometrically on a Licor Odyssey instrument, which is accurate over four orders of magnitude. Size marker, DualColor (BioRad, Hercules, CA, USA). 

### 2.4. Membrane Preparations and CLCA2 Cleavage Experiments

Thirty hours after transfection, cells were scraped from plates and subjected to centrifugation (10,000× *g*, 2 min). Membrane extracts were prepared from cell pellets as described by Bothe, with some modifications [11]. The cell pellets were resuspended in 0.1× PBS, incubated for 10 min at room temperature, and homogenized using a glass pestle in a 2 mL Eppendorf tube. The homogenized mixture was then centrifuged at 500× *g* for 15 min at 4 °C to remove the nuclear pellet, followed by centrifugation of supernatant at 19,700× *g* for 15 min at 4 °C. The membrane pellet was resuspended in PBS, divided into 20 µL aliquots, and supplemented with 1 mM Ca^2+^, Mg^2+^, Mn^2+^, or Zn^2+^ cations or cation-chelator EDTA. The final volume was adjusted to 25 µL with PBS, and membranes were incubated for 30 min or 3 h at 37 °C then subjected to immunoblot analysis. To test whether CLCA2 could cleave mutant protein in the absence of membranes, CLCA-E165Q-Flag protein was immunoprecipitated from HEK293T cell lysates 48 h post-transfection using anti-Flag antibody. The antigen–antibody–bead complex was resuspended in PBS, SDS was added to a final concentration of 0.5%, and the mixture was heated at 95 °C for 5 min. NP40 was added to a final concentration of 9% to inactivate the SDS and allow protein renaturation [20]. Wildtype or CLCA2Δ35 was immunoprecipitated from transfected cell lysates, and the complex was added to the E165Q mixture. ZnCl_2_ was added to a final concentration of 1 mM, and the mixture was incubated for 3 h or 18 h. The percentage of cleaved product was calculated by dividing the fluorometric readings for the product by the sum of product and precursor. 

### 2.5. Co-Transfections and Co-Immunoprecipitation Analysis

pcDNA3.1 (Invitrogen, Carlsbad, CA, USA) or pLex (Thermofisher, Waltham, MS, USA) plasmids expressing wildtype CLCA2 or the E165Q mutant bearing a Flag tag at amino acid 725 [19] were transfected into HEK293T using polyethyleneimine [21]. In some experiments, CLCA2 had a Myc tag inserted at amino acid 33 [22] or no tag as noted. After 48 h, lysates were made in 25 mM Tris pH-7.4, 150 mM NaCl, 2.5 mM MgCl_2_, 0.5 mM EDTA, 0.5% NP40, 1 mM DTT, 5% glycerol, and 1% aprotinin. After confirming similar expression of both proteins by immunoblot, immunoprecipitations were performed with anti-Myc or anti-Flag antibody and Protein A-Sepharose beads (SigmaAldrich, St. Louis, MO, USA) as described [1]. Immunoprecipitates were then subjected to SDS-PAGE, and blots were probed with anti-Myc or anti-Flag antibody.

### 2.6. DNA Sequencing

Sanger DNA sequencing to confirm constructions was conducted by Sigma-Aldrich, Inc., or the Cornell Bioresource Center. Primers were synthesized by Integrated DNA Technologies. Primer sequences are available upon request. 

### 2.7. CLCA2 E165Q Mutation and Δ35 Mutation

The E165Q mutation was introduced into the wildtype CLCA2-Flag construct according to the QuickChangeR (Agilent, Santa Clara, CA, USA) site-directed mutagenesis protocol (Primers: CLCA2E165Q forward 5′-CGAGGCCGAGTGTTTGTCCATCAATG-3′ reverse 5’′CCCAACGGAGGTGGGCCCATTGATG-3′) and was verified by sequencing. CLCA2Δ35 truncates the protein just before the cleavage site. It was constructed by fusing a GST (glutathione-S-transferase) tag to CLCA2 at serine 673. pcDNA3.1-CLCA2 was cut with XhoI, PCR was used to generate a product containing GST with appropriate ends from pGEX-2T (GE Healthcare), the product was inserted into the XhoI site, and the plasmid was sequenced to confirm the absence of mutations. Primer sequences are available upon request.

### 2.8. SOCE Measurements 

HEK293 cells with the E165Q mutation or wildtype CLCA2 were seeded on glass-bottom microwell dishes (MatTek, Ashland, MA, USA) and cultured at 37 °C in a 5% CO_2_ moisturized incubator. When the cells reached 70% confluence, the cells were loaded with a fluorescent Ca^2+^ dye, Fura-2 AM ester (Biotium, San Franscisco, CA, USA), at a concentration of 5 μM in Balanced Salt Solution (BSS) (140 mM NaCl, 2.8 mM KCl, 2 mM MgCl_2_, 10 mM HEPES, pH 7.2) containing 2 mM Ca^2+^ in the dark for 25 min [23]. Then, the cells were washed once with BSS-2 mM Ca^2+^ solution and placed under the fluorescence microscope with a Super Fluo 40× objective (N.A. 0.90, Nikon, Tokyo, Japan) supplemented with a dual-wavelength spectrofluorometer (excitation λ = 350/385 nm and emission λ = 510 nm; Photon Technology International, Birmingham, NJ, USA). The cells were kept in a temperature-controlled moisturized chamber at 37 °C. As previously described [24], the experimental protocol includes two steps: (1) 10 μM thapsigargin (TG) in BSS containing 0.5 mM EGTA was applied for 5 min to deplete ER Ca^2+^ stores; (2) extracellular solution was switched back to BSS-2 mM Ca^2+^ to induce Ca^2+^ influx. The intracellular Ca^2+^ concentration was presented as the ratio of F350 nm/F385 nm, and SOCE was calculated as the changes in the ratio (ΔF350/F385) between the basal level in BSS containing EGTA and thapsigargin (TG/EGTA) and the maximal point after the switch to the BSS-2 mM Ca^2+^ solution (Ca^2+^).

### 2.9. Statistics

Each experiment was repeated at least three times. The results were expressed as means +/− standard deviations (S.D.). Student’s t-test was used for paired or unpaired sample evaluation to assess the statistical significance of the results: * *p* < 0.05, ** *p* < 0.005, and *** *p* < 0.0005. A *p* < 0.05 was accepted as the level of significance. One-way ANOVA was used to test the significance of SOCE measurements.

## 3. Results

### 3.1. CLCA2 Cleavage Is Zinc- and pH-Dependent

**Figure 2:**
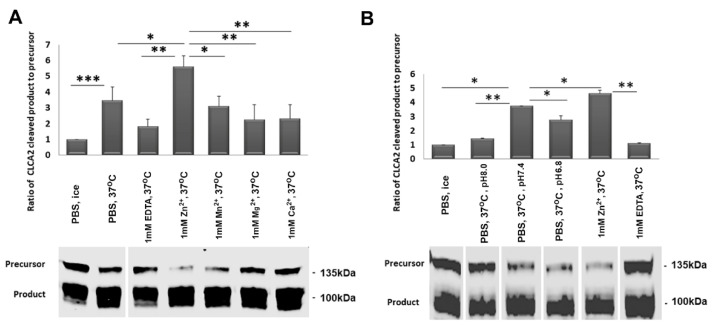
Dependence of cleavage on Zn^2+^ and pH. Immunoblots of crude membrane extracts from CLCA2-transfected HEK293T cells after incubation at 37 °C for 3 h (**A**) in the presence of different cations or EDTA and (**B**) at varying pH values. Input control was incubated on ice. The pH of PBS was 7.4. The fluorometric values of the precursor and cleaved product were determined using a Licor Odyssey scanner. Bar graphs represent the ratio of cleaved product to precursor divided by the value of untreated control in lane one; * *p*-value < 0.05; ** *p*-value < 0.005; *** *p*-value < 0.0005. Blot: TVE20. White vertical lines indicate where lanes were omitted or rearranged to remove irrelevant data. All samples were run on one gel. Experiments were repeated three times.

Previous studies showed that other CLCA family proteins could be cleaved in isolated cell membranes in the presence of certain divalent cations, especially zinc [11,12]. To allow direct comparison between the behavior of CLCA1 and CLCA2, we used the same methodology. First, we isolated crude membranes from HEK293T cells overexpressing CLCA2. After confirming the expression of CLCA2 on cell membranes using an antibody against an N-terminal epitope, we treated the membranes with Zn^2+^, Mg^2+^, Ca^2+^, or Mn^2+^ at 37 °C for 30 min. We found that 1 mM Zn^2+^ increased CLCA2 cleavage compared to untreated membranes or PBS, while the other cations had no effect (Figure 2A). The addition of the metal chelator EDTA reduced cleavage to background levels. These results confirm the prediction that CLCA2 cleavage is dependent on Zn^2+^ [10].

Some cleavage was observed in PBS alone, perhaps due to residual Zn^2+^ bound in vivo. This allowed us to examine the effect of pH on cleavage in the absence of added cations (Figure 2B). A pH of 7.4 produced more cleavage than 6.8 or 8.0. This could be significant because secreted proteins undergo a transition from pH 6.6 in the Golgi and 5.0 in secretory granules to 7.4 at the cell surface. This transition could be a cue for autocleavage.

### 3.2. A point Mutation in the CLCA-N Domain Abolishes CLCA2 Precursor Cleavage 

**Figure 3:**
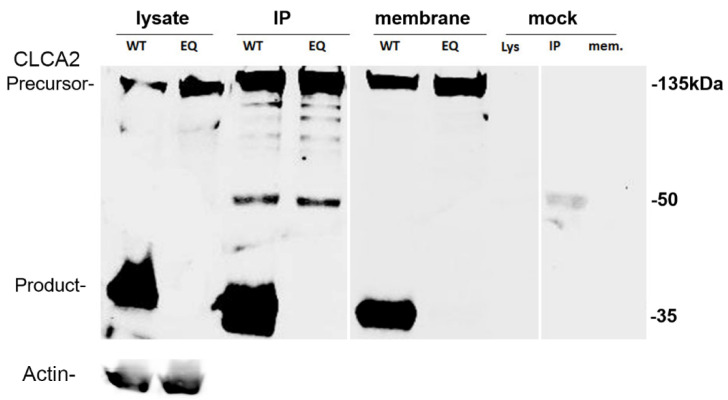
Inhibition of cleavage by E165Q mutation in the HEWAHL amino acid motif of CLCA-N domain. Immunoblot analysis of the wildtype and E165Q mutant forms of Flag-tagged CLCA2 in cell lysates, immunoprecipitates, and membrane extracts from transfected HEK293T cells. Membrane pellets were denatured in SDS sample buffer and loaded directly. Mock refers to cells transfected with the empty pLex vector. Both IP and the blot were with anti-Flag antibody. The band at 50 kDa represents the primary antibody used for IP. WT denotes wildtype CLCA2. EQ denotes the E165Q mutant. Experiment was repeated at least three times.

The HEWAHL sequence is the metal-binding motif present in the CLCA-N domain of CLCA2. The glutamate (E) is vital for zinc-binding in other Zn^2+^-dependent metalloproteases, and its mutation in other CLCA family members abolished or severely reduced cleavage [11,12,13]. To determine this for CLCA2, we changed glutamate (E) to glutamine (Q) in this active site (E165Q). In cell lysates, immunoprecipitates, and isolated cell membranes, this mutation completely abolished CLCA2 cleavage (Figure 3). These results indicate that the glutamate at aa165 is essential for CLCA2 cleavage and suggest that the CLCA2 metalloprotease domain is responsible.

### 3.3. Concentration Dependence Suggests Trans Cleavage

**Figure 4:**
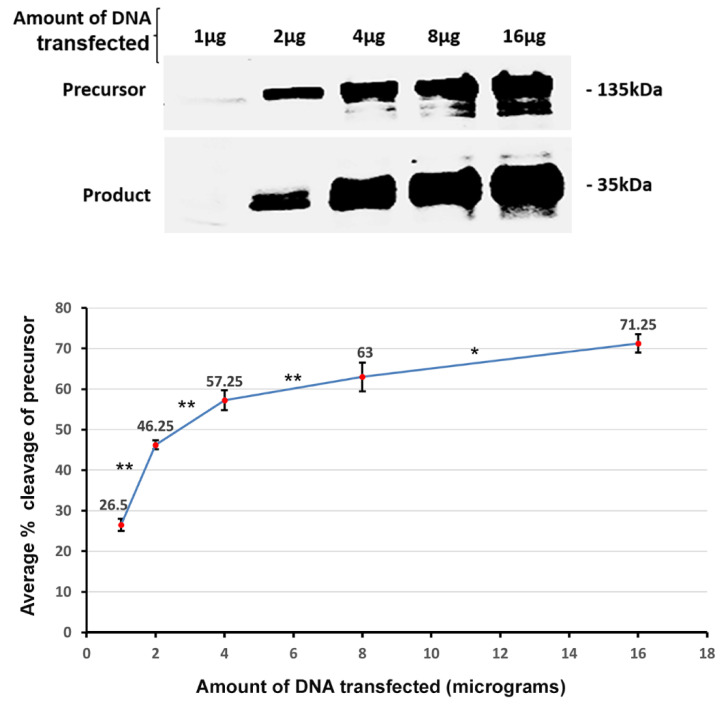
Effect of substrate concentration on cleavage efficiency. Top, immunoblot analysis of CLCA2-Flag cleavage from lysates of HEK293T cells transfected with increasing amounts of DNA. Bottom, graph relating percent cleavage to quantity of DNA transfected. Percent cleavage was obtained by dividing the signal for product by the sum of signals for product and precursor. Experiment was repeated four times; * *p*-value < 0.05; ** *p*-value < 0.005. Blot, anti-Flag.

Conceivably, a single CLCA2 molecule might cleave itself (cis), or cleavage might be intermolecular (trans). We expected that the efficiency of a trans cleavage event should vary with the concentration of the reactants, whereas the efficiency of cis cleavage should not. To test this, we transfected HEK293T cells with increasing amounts of CLCA2. We found that as the concentration of the precursor molecules increased, there was an increase in the percent cleavage of CLCA2 from 26.5 to 63 (Figure 4). This effect is consistent with trans cleavage, not cis. It is also inconsistent with the cleavage of CLCA2 by a different metalloprotease. In that case, an increase in template concentration would cause a decline in the cleavage efficiency due to the metalloprotease becoming the limiting factor.

### 3.4. Cleavage of E165Q Mutant in Trans by Wildtype CLCA2

**Figure 5:**
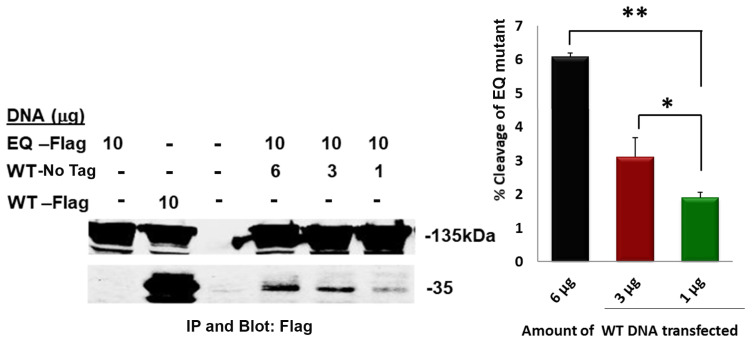
The E165Q mutant can be cleaved by co-transfected wildtype CLCA2 in a concentration-dependent manner. Left, immunoblot analysis of the co-transfected wildtype (WT) and E165Q (EQ) mutant anti-Flag immunoprecipitates from 1% NP40 lysates. Cleavage of the mutant protein is indicated by the 35 kDa band in lanes 4–6. Lanes 1–2 show level of cleavage product from cells transfected with EQ alone or WT alone; lane 3, nontransfected. Right, relation between percent cleavage of the EQ and amount of co-transfected wildtype CLCA2; * *p*-value < 0.05; ** *p*-value < 0.005. The percent cleavage of WT-Flag in lane 2 is 55. Experiment repeated three times.

To further address this notion, we co-transfected different quantities of untagged wildtype CLCA2 with the Flag-tagged E165Q mutant and compared the level of 35 kDa product with that of singly transfected Flag-tagged controls (Figure 5A, lanes 1 and 2, EQ and WT). While 0% of the E165Q control and 55% of the wildtype control were cleaved, 1.5 to 6% of the co-transfected mutant protein was cleaved, and cleavage was proportional to the amount of co-transfected wildtype CLCA2 (Figure 5A, lanes 4–6). These results are only consistent with intermolecular endoproteolysis.

### 3.5. CLCA2 Precursors Interact to Form a Stable Complex

**Figure 6:**
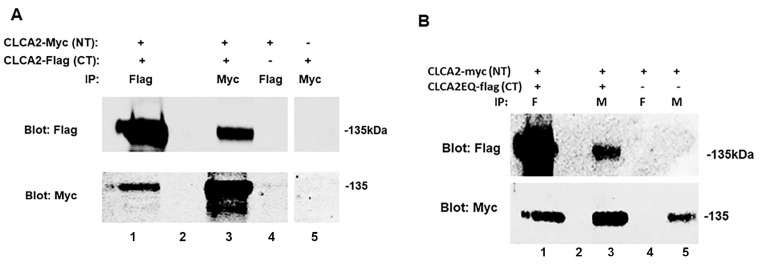
Co-immunoprecipitation of CLCA2 precursors. (**A**) Immunoblots of lysates from co-transfected or singly transfected HEK293T cells that were subjected to immunoprecipitation with anti-Flag or anti-Myc antibodies. NT indicates N-terminal insertion of the Myc tag. CT indicates C-terminal insertion of the Flag tag. The Myc antibody immunoprecipitates only the Flag-tagged precursor but not the C-terminal product. (**B**) E165Q mutation does not affect ability to form a stable complex. Immunoblot analysis of co-immunoprecipitation from lysates of HEK293T cells transfected with Myc-tagged wildtype CLCA2 and/or Flag-tagged CLCA2 E165Q mutant. On both gels, lane 2 was left empty to avoid spillover. Experiments were repeated three times.

In light of the evidence that cleavage occurs in trans, we hypothesized that CLCA2 precursors interact to form a stable complex. We tested this by co-transfecting CLCA2-Myc with CLCA2-Flag (see Figure 1) and testing whether they co-immunoprecipitated. We found that antibodies against either tag immunoprecipitated both precursor molecules (Figure 6A, lanes 1 and 3). Controls in lanes 4 and 5 confirmed the specificity of the antibodies for their respective epitope tag. 

### 3.6. E165Q Mutation Does Not Affect the Ability to Form a Stable Complex

To address the possibility that E165Q mutation might inhibit cleavage by blocking complex formation, we co-transfected the Flag-tagged mutant with Myc-tagged wildtype CLCA2 and tested their ability to co-immunoprecipitate. The mutant protein co-immunoprecipitated with the wildtype (Figure 6B, lanes 1 and 3), similar to results of the previous experiment.

### 3.7. Autocleavage by Immunopurified CLCA2 Requires Denaturation/Renaturation

**Figure 7:**
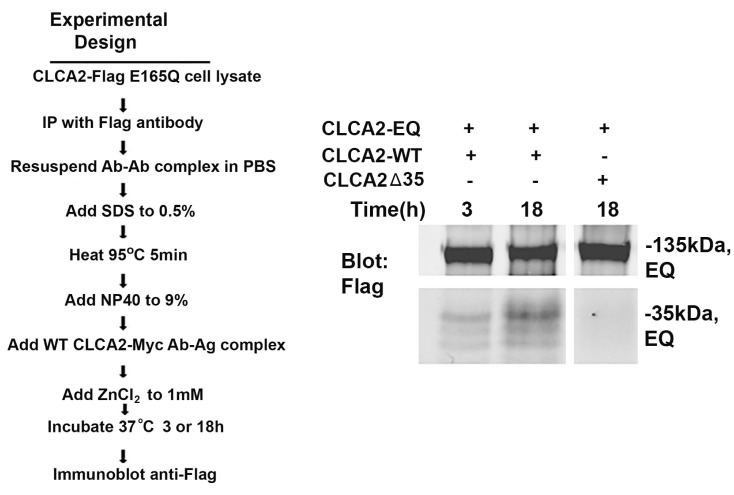
Cleavage of immunopurified E165Q mutant (EQ) by immunopurified wildtype CLCA2 (WT) in vitro after denaturation of the mutant protein by SDS. Left, experimental flowchart. Right, immunoblot analysis of cleavage products using the anti-Flag antibody. CLCA2-WT and CLCA2Δ35 lacked a Flag-tag; bands represent the EQ mutant precursor and products. Incubation time is indicated. The lower panel was overexposed relative to the upper panel to generate visible bands. Experiment repeated three times.

Cleavage experiments in crude membranes could not conclusively rule out the involvement of other metalloproteases. Therefore, we tested whether immunopurified proteins were capable of self-cleavage in the absence of membranes. Initial attempts failed to detect any cleavage, suggesting that membrane anchorage might provide conformational information essential for cleavage. We reasoned that denaturation by SDS followed by slow renaturation in NP40 might relieve any conformational constraints in the target molecule (Figure 7). When the purified E165Q mutant protein was so treated and mixed with wildtype CLCA2 in the presence of 1 mM Zn^2+^, cleavage was evident by 3 h and increased through the 18 h duration of the experiment (Figure 7, lanes 1 and 2), while no cleavage of E165Q was observed when mixed with a deletant of CLCA2 truncated at the cleavage site (i.e., CLCA2Δ35; Figure 7, lane 3). These results suggest that the cleavage site at amino acid 708 is inaccessible in the absence of membrane anchorage.

### 3.8. CLCA2 Ectodomain Undergoes Conformational Shift upon Cleavage

**Figure 8:**
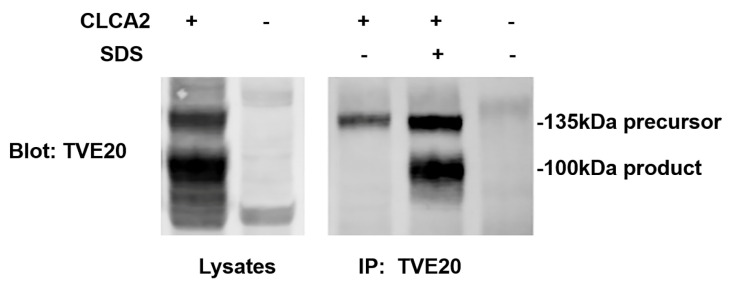
Evidence that the N-terminal ectodomain undergoes conformational shift upon cleavage that masks an epitope. Lanes 1 and 2, detection of precursor and N-terminal product on SDS-PAGE immunoblot of HEK293 NP40 lysates. Lane 3, immunoprecipitation of precursor only from NP40 lysate. Lane 4, detection of precursor and product after lysate was first denatured by SDS/heat then renatured by addition of excess NP40 as in Figure 7. Lanes 2 and 5, HEK293 expressing vector only. Experiment repeated three times.

We generated an antibody against amino acids 643–663, termed TVE20, just N-terminal to the cleavage site at 708 (see Figure 1). We observed unexpectedly that, although this antibody detected both the 135 kDa precursor and the 100 kDa amino-terminal product on immunoblots of cell lysate, it immunoprecipitated only the precursor, suggesting that the region containing its epitope became inaccessible following cleavage (Figure 8, lanes 1 and 3). To test this idea, we subjected cell lysate containing CLCA2 to the same denaturation–renaturation procedure as in Figure 7 and then immunoprecipitated it with TVE20. This procedure restored recognition of the epitope and immunoprecipitation (Figure 8, lanes 3 and 4). These results suggest that cleavage of CLCA2 induces a conformational shift that may change the activity of the protein. 

### 3.9. E165Q Abolishes SOCE Stimulation by CLCA2

**Figure 9:**
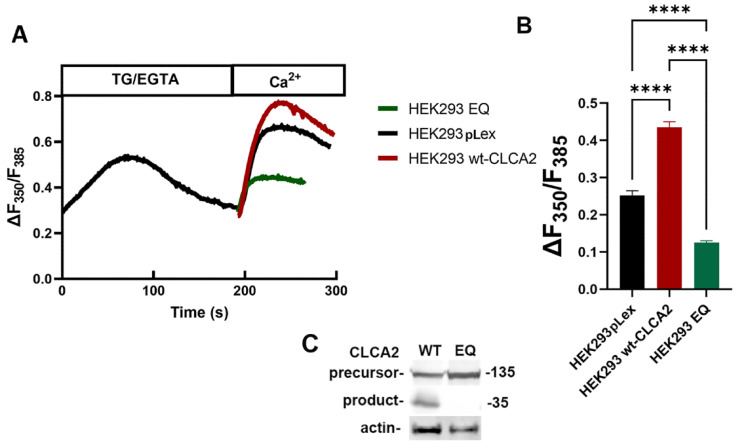
Reduced SOCE in the cells expressing CLCA2 E165Q mutant. (**A**) Representative traces of intracellular Ca^2+^ in pLex vector control (HEK293 pLex), wildtype CLCA2 (wt-CLCA2), and E165Q mutant (HEK293-EQ) cells. (**B**) SOCE activity was calculated as the changes in the ratio of F350 nm/F385 nm (ΔF350/F385). Statistical analyses were vehicle control (0.25 ± 0.089) and wildtype CLCA2 (0.44 ± 0.125) or E165Q mutation (0.12 ± 0.037). Mean ± SD, *n* > 30 cells, ****: *p* < 0.0001 (based on one-way ANOVA). Experiment was repeated three times. (**C**) Immunoblot of wildtype (WT) and E165Q (EQ) cell lines used in the analysis. Blot: anti-Flag and anti-actin antibodies.

To investigate the physiological significance of the CLCA2 metalloprotease activity, we tested its effect on the ability of CLCA2 to activate SOCE in response to the depletion of ER Ca^2+^ reserves. In our previous study, we established that CLCA2 interacts with and co-localizes with the Orai1–STIM1 complex on the plasma membrane that regulates SOCE in response to the depletion of ER Ca^2+^ reserves; that ectopic expression of CLCA2 in HEK293 (which has no endogenous CLCA2) enhances ER Ca^2+^ release and SOCE; and that CLCA2 knockdown in mammary epithelial cells reduces both [7]. Here, using the Fura-2 ratiometric method, we first confirmed our previous observation that wildtype CLCA2 enhanced the SOCE response compared to the vector control (Figure 9A,B). In contrast, cells expressing the E165Q mutant protein had substantially less SOCE even than the vector control, suggesting a dominant-negative effect.

## 4. Discussion

A hallmark of the CLCA protein family is its cleavage near amino acid 700 [25,26]. We previously demonstrated that, in contrast to other family members, the CLCA2 precursor remains intact while translocating to the plasma membrane where it is cleaved to yield an N-terminal ectodomain and a membrane-anchored C-terminal domain [1]. Neither the stimulus for cleavage nor the cleaving agent were identified; nor was a functional consequence of the cleavage determined. 

To understand the significance of the stress-induced cleavage to CLCA2 function, we investigated the cleavage event, taking advantage of the large body of work on the self-cleavage of other family members, particularly CLCA1 [11,12,13]. Using isolated membranes from transfected cells, we determined that cleavage depends on zinc and pH and that mutation of the HEWAHL metal-binding motif abolishes cleavage. The dependence on zinc echoes previous findings with CLCA1 [11] and further confirms the prediction of Pawloski based on structural modeling that CLCA proteins are zincins and that the glutamate is important for this function [10,11]. The preference for a neutral pH could be significant, because secreted proteins undergo a transition from pH 6.6 in the Golgi and 5.0 in secretory granules to 7.4 at the cell surface. This transition could be a cue for the autocleavage of CLCA2 at the cell surface. The self-cleavage of other CLCA family members in the ER or early Golgi might be explained by sequence differences leading to different pH preferences.

**Figure 10:**
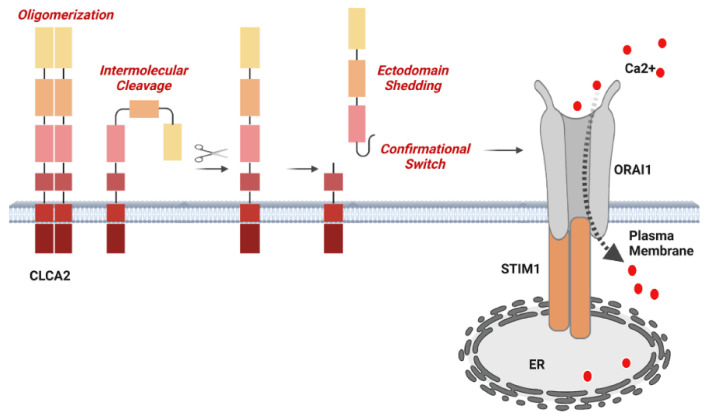
Model showing that CLCA2 forms complexes, that cleavage is intermolecular and causes a conformational switch, and that the cleavage activity is necessary for activation of SOCE. The stoichiometry is unknown and is depicted here as a dimer for simplicity. Both the precursor and cleaved forms have been shown to interact with the Orai–STIM complex, but structural details are unknown.

In addition, we found that cleavage efficiency depended on the amount of protein expressed, suggesting intermolecular cleavage. This was demonstrated by the co-immunoprecipitation and cleavage of the mutant protein by co-transfected wildtype CLCA2. We found that this cleavage could not be reproduced with immunopurified proteins unless they were first denatured and renatured, suggesting that cleavage depends on a factor within the membrane or a conformation induced by membrane insertion. Serendipitously, we discovered evidence that the cleavage induces a conformational shift in the ectodomain: an antibody with an epitope in the ectodomain immunoprecipitated the CLCA2 precursor but not the cleaved ectodomain unless it was first denatured and renatured. Together, these results led us to test whether the mutant lacking cleavage activity had lost the ability to regulate SOCE. A model based on these results is presented in Figure 10.

Some of the results reported here echo findings with CLCA1, while others are distinct. A recent report suggests that the split between CLCA1 and CLCA2 is ancient, as avian species have only these two genes, and other family members in mammals appear to be derived from CLCA1 [27,28]. In all mammalian species studied, CLCA1 is a fully secreted protein, while CLCA2 is an integral membrane protein [1,29]. Both are zinc-dependent and cleave intermolecularly but differ in dependence on cell membranes. CLCA1 self-cleaved in the absence of membranes without the need for the denaturation–renaturation step required here [13]. It should be noted that the amino acid sequence of the transmembrane segment of CLCA2 is highly conserved in mammals and has been shown to mediate the interaction between CLCA2 and a cell–cell adhesion protein [30]. Thus, it may be expected to play a role in its translocation and activation. We also found that CLCA2 is capable of self-associating into immunoprecipitable complexes but did not investigate the stoichiometry. A recent structural study of CLCA1 found that it assembles into high-molecular-weight oligomers consistent with an octamer [31]. Such studies have not yet been published for CLCA2.

The best evidence that the cleavage of CLCA2 is functionally significant is our discovery that the E165Q mutation not only abolishes the enhancement of SOCE by CLCA2 but reduces it to less than half the level seen in the vector control. Previous immunoprecipitation studies detected both precursor and cleaved product in this complex [7]. The present results suggest that the precursor acts negatively on the Orai–STIM complex, while cleavage activates it. The finding that cleavage causes a conformational shift in the N-terminal CLCA2 product suggests that cleavage may trigger a corresponding shift in the channel complex resulting in increased open probability or complex stability. Further studies are needed to determine what aspect of the channel is affected. Given the role of intracellular calcium levels in many physiological and pathological processes and the role of CLCA2 in differentiation and stress response, these findings may provide a means for manipulating SOCE therapeutically. 

## 5. Conclusions

Based on the present data, we conclude that human CLCA2 has zinc-dependent metalloprotease activity that is required for its physiological function in regulating intracellular calcium. We found that self-cleavage produces a conformational shift. Further molecular and biochemical studies are needed to determine how this activates the Orai1 calcium channel. Given the regulation of CLCA2 expression by p53 and differentiative signaling, it will be interesting to investigate whether these pathways also regulate self-cleavage of the protein.

## Data Availability

Not applicable.
